# Long-term clinical outcomes of patients with sympathetic ophthalmia

**DOI:** 10.1007/s10792-024-03007-x

**Published:** 2024-02-07

**Authors:** Neofytos Mavris, Radgonde Amer

**Affiliations:** Department of Ophthalmology, Hadassah Medical Center, POB 12000, 91120 Jerusalem, Israel

**Keywords:** Dalen-Fuchs nodules, Granulomatous uveitis, Ocular trauma, Sympathetic ophthalmia

## Abstract

**Purpose:**

To present the long-term clinical outcomes of patients with sympathetic ophthalmia (SO).

**Methods:**

Retrospective review of patients’ medical files between 2002 and 2022.

**Results:**

Included were seven patients (four males). The mean ± SD age at presentation was 37.9 ± 22.5 years. Four patients had co-morbidities: three had diabetes mellitus type 2 and one had Turner Syndrome. Trauma was the inciting event in six patients and postoperative endophthalmitis in one patient. Decreased visual acuity (VA) was the leading symptom in the sympathizing eye and all of the patients presented with panuveitis. The mean ± SD interval between the triggering incident and the onset of SO in six cases was 4.3 ± 4.2 months. One case presented 30 years following the eye injury. Five patients underwent enucleation/evisceration of the exciting eye. The mean ± SD presenting LogMAR BCVA in the sympathizing eye was 0.57 ± 0.82, and the final LogMAR BCVA was 0.61 ± 0.95. Inflammation was completely controlled in 5 patients at a mean ± SD of 8.55 ± 9.21 months following the institution of immunomodulatory therapy, and it was partially controlled in 2 patients. VA deteriorated in all 3 diabetic patients and improved or remained stable in the 4 young and healthy patients. The mean ± SD follow-up period after achieving drug-free remission was 28 ± 22.8 months. The mean ± SD follow-up time was 6.8 ± 5.6 years.

**Conclusions:**

SO is one of the most sight-threatening conditions, affecting the healthy eye. In this cohort, the favorable visual outcome was especially seen in young and healthy individuals. Visual prognosis is directly related to prompt diagnosis and treatment.

## Introduction

Sympathetic ophthalmia (SO) is an inflammatory disease characterized by bilateral granulomatous uveitis [1], occurring after a penetrating ocular injury (0.19%) [2] or intraocular surgery (0.01%) [3]. An autoimmune response is believed to arise when uveal antigens are exposed to Langerhans cells, which are present in the conjunctiva [4]. The traumatized or operated eye is considered the exciting eye, and the fellow contralateral eye is the sympathizing one [5].

The theory was first suggested by Hippocrates [6], but it was not until 1840 that Mackenzie coined the term Sympathetic Ophthalmia [7]. It is a rare disease, with an estimated yearly incidence rate after open globe injury of 33 per 100,000 persons (95% CI 19.61–56.64) [2].

The onset time varies; patients can present with decreased vision, pain, or floaters in the sympathizing eye between 5 days [8] and 66 years [9] following the incident. Most cases (90%) arise within the first year of injury/surgery [10]. The clinical signs can also vary, and they may include mutton-fat keratic precipitates, exudative retinal detachment (ERD), and Dalen-Fuchs (DF) nodules [3] which are the characteristic pathological finding of SO [11]. Optical coherence tomography (OCT) can be used to indicate the presence of DF nodules, monitor macular edema, and follow the disease’s progression [1].

We aim to present the long-term clinical outcomes of seven patients with sympathetic ophthalmia evaluated between 2002 and 2022.

## Methods

### Study design

This retrospective single-center study reviewed the medical records of patients with SO who were diagnosed and managed at the Department of ophthalmology of Hadassah Medical Center, a tertiary-care hospital, with at least one year of follow-up. Approval for proceeding with the study was received from the Ethics Review Committee of Hadassah Medical Center (No. 0575-12-HMO). The diagnosis of SO was clinical, based on a definite history of ocular surgery or injury and subsequent bilateral granulomatous uveitis. Other causes of ocular inflammation, like tuberculosis, sarcoidosis, and syphilis, were excluded through laboratory and imaging examinations. In addition, ocular imaging was performed, including OCT (Zeiss Stratus OCT, Carl Zeiss Meditec, Dublin, CA, USA, and Heidelberg Spectralis OCT, Heidelberg Engineering, Heidelberg, Germany), color fundus photography (Optos Silverstone swept-source OCT, Optos PLC, Dunfermline, UK and TRC-50DX, Topcon Corporation/Kabushiki-gaisha Topukon, Tokyo, Japan) and fundus fluorescein angiography (FFA) (Optos Silverstone swept-source OCT, Optos PLC, Dunfermline, UK and TRC-50DX, Topcon Corporation/Kabushiki-gaisha Topukon, Tokyo, Japan).

### Data collection

Demographic and clinical data of patients with SO was collected from the database of the uveitis service of Hadassah Medical Center. This included age at presentation, gender, ocular and systemic background, history of triggering event, symptoms, the time interval between the triggering event and the onset of SO, presenting signs, duration of active inflammation, immunosuppressive therapy, surgical procedures and secondary complications. Standardization of uveitis nomenclature (SUN) criteria was used to describe and classify the anatomical type of uveitis [12]. Best-corrected visual acuity (BCVA) was assessed at the presentation, during follow-up examinations and at the patients’ last visit to our institute using the Snellen chart. LogMAR (logarithm of the minimum angle of resolution) notation was used to compute the change in visual acuity (VA).

### Outcome measures

Clinical outcomes included the initial and final BCVA, duration between onset of symptoms and control of inflammation, exacerbations, enucleation or evisceration of the inciting eye, and secondary complications. In the study, VA was deemed to have worsened if the final BCVA dropped by at least two lines on the Snellen chart from the initial BCVA. BCVA was considered stable if no alteration was observed between the starting and final measurements, while improvement was acknowledged when the Snellen chart revealed an increase of at least two lines from the initial to the final BCVA. The VA was evaluated for any significant changes across various timelines using Friedman’s ANOVA test. We considered a *p*-value of 0.05 as the threshold for statistical significance.

## Results

### Demographic and clinical data

Seven patients (4 males) with a mean ± SD age at presentation of 37.9 ± 22.5 years were included. Three patients had Diabetes Mellitus (DM) type 2, and one had Turner syndrome. Trauma was the inciting event in 6 patients, and complicated intraocular surgery with postoperative endophthalmitis in one patient (Table 1). The mean time interval ± SD between the triggering event and the onset of SO was 55.11 ± 134.5 months. One patient developed SO 30 years after the eye trauma. By excluding this patient, the mean time interval became 4.3 ± 4.2 months. Decreased VA was the leading symptom (5 eyes out of 7, 71%) in the sympathizing eye. Clinically, 6 out of 7 patients presented with signs of bilateral panuveitis, and one patient (#4) presented with panuveitis in the exciting eye and signs of anterior uveitis in the sympathizing eye. Regarding the sympathizing eyes, anterior chamber (AC) cells were noted in 7 eyes (100%), vitreous cells in 6 eyes (86%), papillitis in 4 eyes (57%), DF nodules in 4 eyes (57%), ERD in 2 eyes (29%) and cystoid macular edema (CME) in 1 case (14%).

**Table 1:** Demographics and clinical data of patients with sympathetic ophthalmia (SO) at presentation

Patient	1	2	3	4	5	6	7
Age/gender	25M	12M	19M	34F	57F	42F	76M
Co-morbidities	No	No	No	Turner syndrome	DM, HTN, obese	DM, HTN, obese	DM
*Triggering event*							
Trauma	RE	RE	RE	RE	LE	LE	
Complicated surgery							RE
Time interval between inciting event and onset of SO	3 months	6 weeks	2 months	5 weeks	6 months	30 years	1 year
Initial BCVA	RE NLP LE 20/62	RE NLP LE 20/22	RE NLP LE 20/33	RE 20/50 LE 20/50	RE 20/40 LE NLP	RE 20/28 LE NLP	RE NLP LE HM
Symptoms (SE)	Decreased vision	Photophobia, redness, pain	–	Decreased vision, redness, headache	Decreased Vision	Decreased vision, redness	Decreased vision
*Signs* (SE)							
Keratic precipitates	No	Dust-like	Mutton-Fat	Mutton-Fat	Mutton-Fat	Dust-like	Mutton Fat
Anterior chamber cells/flare	Yes	Yes	Yes	Yes	Yes	Yes	Yes
Vitreous cells	Yes	Yes	Yes	No	Yes	Yes	Yes
Papillitis	No	No	Yes	No	Yes	Yes	Yes
Macular edema	No	No	No	No	No	No	Yes
Exudative Retinal detachment	Yes	Yes	No	No	No	No	No
Dalen Fuchs nodules	No	Yes	No	No	Yes	Yes	Yes

*DM* Diabetes mellitus type 2, *HTN* Hypertension, *RE* Right eye, *LE* Left eye, *BCVA* Best corrected visual acuity, *NLP* No light perception, *HM* Hand motion, S*E* Sympathizing eye

Examination and follow-up were carried out by using OCT in 6 patients and FFA in 4 patients.

### Management and exacerbations

The mean (± SD) follow-up time was 6.8 ± 5.6 years. Five patients were treated with dual immunosuppression with systemic corticosteroids and corticosteroid-sparing agents **(**Table 2**)**. One patient was treated with triple immunosuppression, and one patient with systemic corticosteroids only. The mean (± SD) of the total duration of immunomodulatory therapy (IMT) (steroid-sparing) was 67.3 ± 78.8 months. Topical corticosteroids were given as needed. Enucleation/evisceration was performed in 2 patients within 2 weeks of SO onset and in 3 patients between 1 month and 2 years after SO onset (Table 2**)**.

**Table 2:** Treatment and outcomes of patients with sympathetic ophthalmia (SO)

Patient	1	2	3	4	5	6	7
Treatment	CS, MTX	CS, MTX	CS	CS, Aza	CS, TNF-i, MMF*	CS, MTX	CS, MMF
Total duration of IMT (steroid-sparing) (months)	42	216	-	26	72	36	12
Interval between SO onset and EN/ EV of exciting eye	RE EN 84 weeks	RE EN 1 week	RE EN 4 weeks	No	LE EV 2 days	LE EN 32 weeks	No
Time till control of SO inflammation (months)	10	3.25	4	1.5	24	Partial control	Partial control
Follow up (months)	48	228	84	66	72	36	36
Follow up period after drug-free remission (months)	6	12	54	40	–	–	–
Final BCVA	LE 20/20	LE 20/22	LE 20/28	RE 20/50 LE 20/50	RE 20/100	RE 20/40	RE NLP LE LP
Complications (sympathizing eye)	Choroidal scars	Choroidal scars	Choroidal scars	Choroidal scars, macular pigment changes	Choroidal scars, maculopathy, PS	PSC, choroidal scars, maculopathy, PS	Choroidal scars, rubeosis, cataract, PS

*CS* Corticosteroids, *MTX* Methotrexate, *TNF-i* Tumor necrosis factor inhibitors (adalimumab, infliximab), *MMF* Mycophenolate mofetil, *Aza* Azathioprine, *IMT* Immunomodulatory therapy, *EN* Enucleation, *EV* Evisceration, *PSC* Posterior subcapsular cataract, *PS* posterior synechiae

^*^patient’s left eye was first injured in childhood that resulted in blindness

Inflammation was completely controlled in 5 patients at a mean (± SD) of 8.55 ± 9.21 months following the institution of IMT, and it was partially controlled in 2 patients. During follow-up, 5 patients (71%) experienced disease exacerbation: in two patients (pt#2 and 4), recurrence occurred 10 weeks and 2 years after treatment cessation; in two patients (pt#5 and 7), exacerbation occurred 5 and 24 months after disease onset while on maintenance treatment with IMT and systemic steroids. One case (pt#6) experienced exacerbation shortly after every attempt to decrease steroid treatment. Exacerbation was controlled in 3 out of those 5 cases who suffered from relapses, while 2 patients had persistent uveitis. Four patients (pt# 1–4) attained drug-free remission. The mean (± SD) follow-up period after achieving drug-free remission was 28 ± 22.8 months.

### Visual acuity

**Fig. 1:**
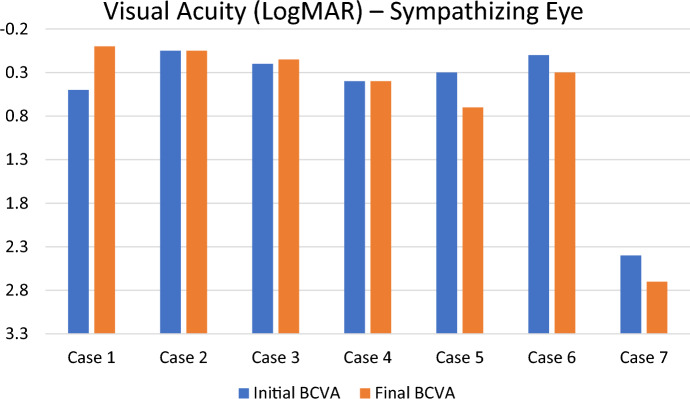
Initial and final Best Corrected Visual Acuity (LogMAR) of the sympathizing eye

The mean (± SD) presenting LogMAR BCVA in the sympathizing eyes was 0.57 ± 0.82. The mean (± SD) LogMAR BCVA was 0.51 ± 0.88, 0.54 ± 0.85, and 0.63 ± 1.05 at one, two, and 3 years later, respectively. The final mean (± SD) LogMAR BCVA was 0.61 ± 0.95 (Tables 1, 2). There was no significant change in VA at years 1 (*p* = 0.102), 2 (*p* = 0.655), 3 (*p* = 1.0), and final VA (*p* = 0.655) when compared to the initial VA. Four young and healthy patients (pt# 1–4) exhibited improved or stable visual outcome in the sympathizing eye **(**Fig. 1**).** However, deterioration in VA was observed in all three diabetic patients (pt# 5–7).

### Complications

With regard to ocular complications in the sympathizing eye, peripheral choroidal atrophic lesions were noted in all 7 patients (pt#1–7), cataracts in 2 patients (pt#6, 7), posterior synechiae in 3 patients (pt#5–7), maculopathy in 2 cases (pt#5, 6), macular pigmentary changes in 1 patient (pt#4), and rubeosis in 1 patient (pt#7).

Case 1

A 25-year-old healthy male patient presented with right eye (RE) scleral perforation following blunt trauma. The patient underwent scleral suturing, lensectomy, vitrectomy, and 360°- encircling buckle. Postoperatively, RE VA was no light perception (NLP), and it was 20/20 in left eye (LE). LE anterior and posterior segments were normal.

**Fig. 2:**
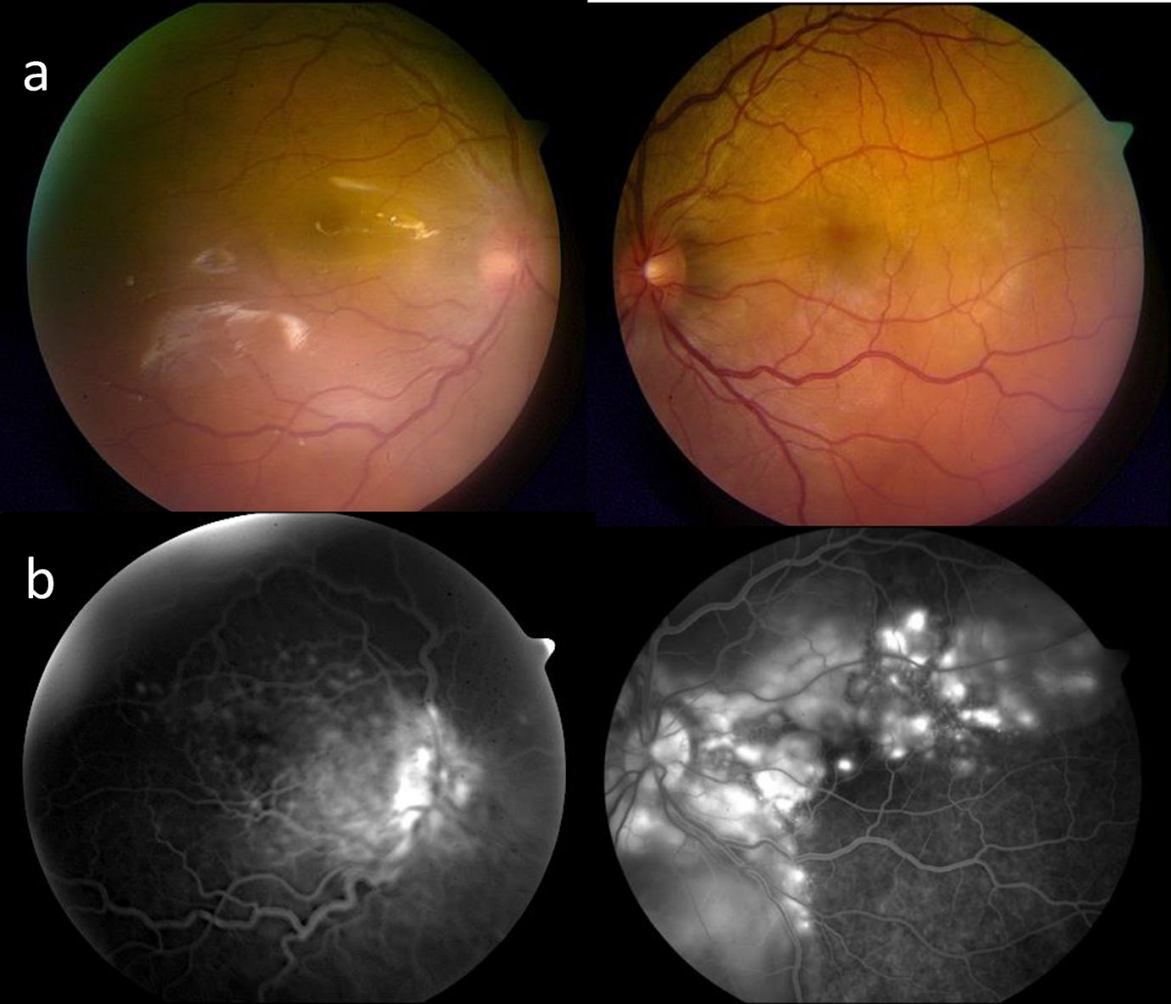
**a** Colour fundus photos of both eyes. A hazy fundus view and blurred margins of optic disc are noted in the right eye, left image. Blunt foveal reflex is noted in the left fundus, right image, with faint gray subretinal lesions temporal to fovea. **b** Fluorescein angiogram of both eyes. Late leakage around and at the optic discs is seen bilaterally. Hyperfluorescent areas of pooling compatible with exudative retinal detachment are noted in the left fundus, right image

**Fig. 3:**
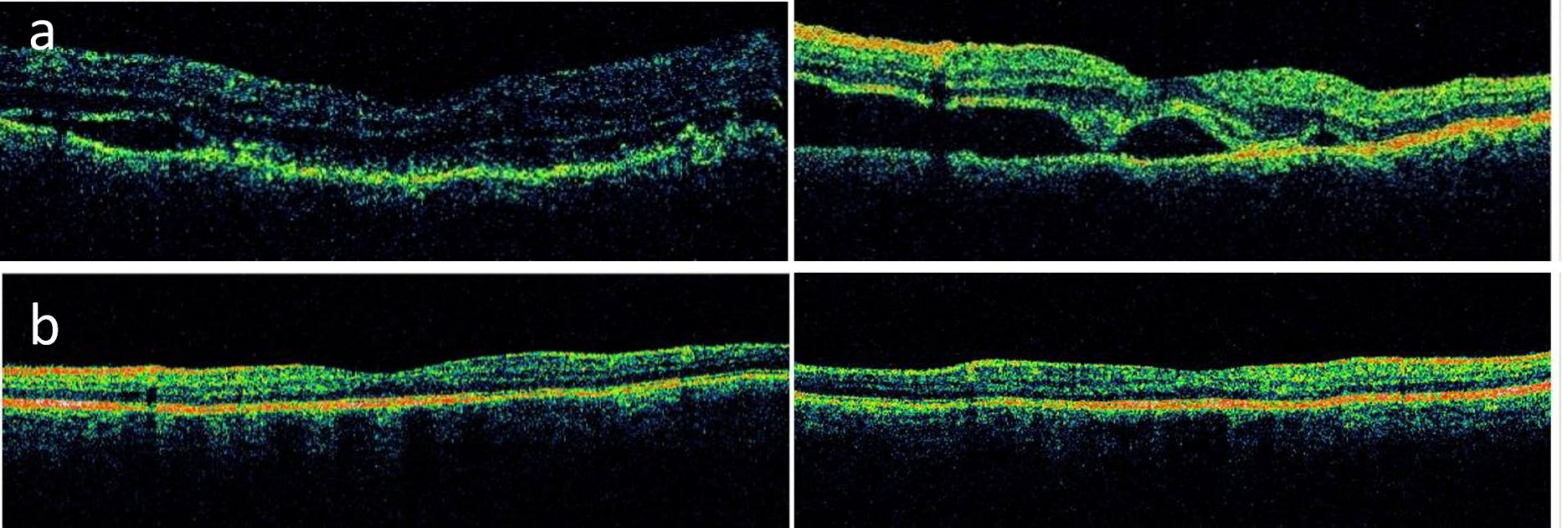
**a** OCT of both eyes. On the upper left side is the traumatized, exciting right eye, with subretinal fluid; on the upper right, the sympathizing left eye has exudative retinal detachment. **b** OCT of the exciting (lower left image) and sympathizing (lower right image) eyes showing complete resolution of subretinal fluid after the institution of systemic corticosteroids

3 months later, the patient presented with a sudden decrease in LE VA. LE VA was 20/62. Bilateral panuveitis **(**Fig. 2a**)** with ERD was diagnosed and confirmed on OCT (Fig. 3a). FFA showed several foci of hyperfluorescence in the posterior poles, bilateral pooling and optic disc hyperfluorescence (Fig. 2b).

Sympathetic ophthalmia was diagnosed and the patient was treated with prednisone and methotrexate (MTX). Control of inflammation was achieved by the 10th month of therapy, with complete resolution of ERD (Fig. 3b).

The patient initially declined enucleation of the exciting eye. However, band keratopathy developed and subsequently RE became painful. Therefore, RE enucleation was performed 2 years after the trauma. Medical therapy with MTX and prednisone was discontinued 3.5 years after the trauma. On his last follow-up, 4 years after the triggering event, LE VA was 20/20.

Case 2

A 12-year-old boy presented to the emergency room complaining of LE photophobia, redness, and pain of a 1-week duration. 6 weeks earlier, the patient underwent twice RE intraocular surgeries due to a perforating injury, which included primary repair of the wound, lensectomy, vitrectomy, retinotomy, endolaser, and silicone oil tamponade.

**Fig. 4:**
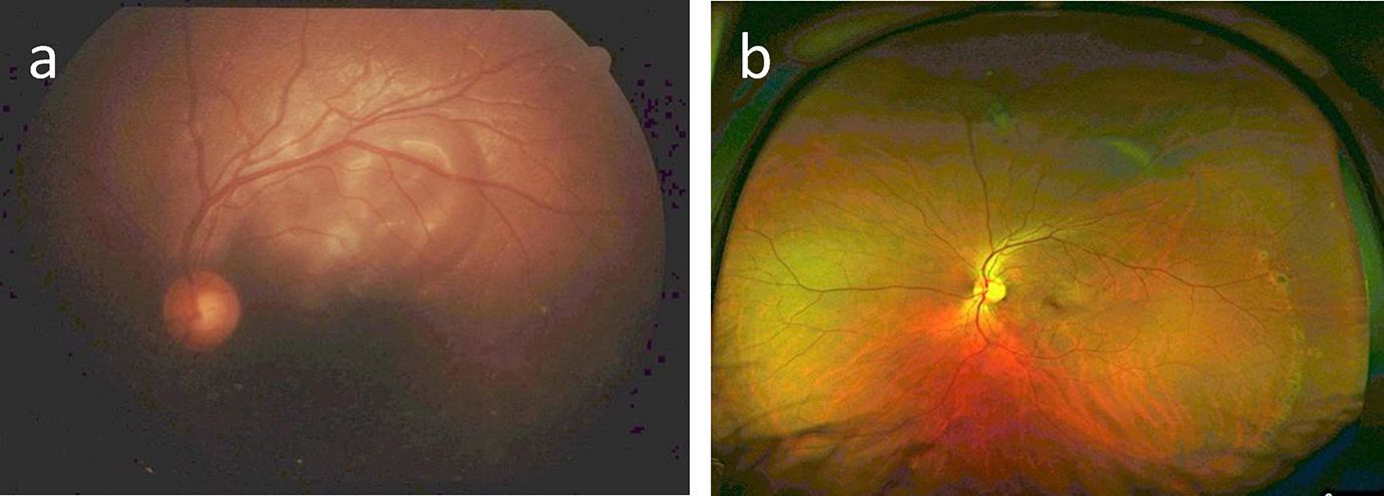
**a** Color fundus picture of the LE showing exudative retinal detachment along the superotemporal arcade. **b** A pseudocolor fundus photograph of the LE shows clear vitreous, normal optic disc and inferotemporal peripheral choroidal pigmentary lesions

BCVA was NLP in RE and 20/20 in LE. There was a big corneal scar in RE with band keratopathy and conjunctival injection. Panuveitis was diagnosed in LE with non-granulomatous anterior uveitis, vitritis, choroiditis and ERD in the macular area (Fig. 4a).

SO was diagnosed and prednisone was instituted. MTX was subsequently added as a steroid-sparing agent. RE enucleation was performed within the first week of SO onset. Choroiditis completely resolved in the first week.

10 weeks later, the patient stopped all medications which has led to uveitis exacerbation. Medications were resumed and a complete resolution of the inflammation was achieved 3 weeks later. LE showed a quiet anterior segment, clear vitreous, peripheral choroidal pigmentary lesions (Fig. 4b), and resolution of ERD. BCVA of LE was 20/20, and since then, the eye has remained quiet and stable on maintenance treatment with MTX.

18 years later, IMT was discontinued. One year later, LE final BCVA remained 20/22 with no signs of inflammation.

Case 3

A 19-year-old healthy male patient sustained RE penetrating injury, for which he underwent primary repair of the sclera, lensectomy, and vitrectomy with silicone oil tamponade due to retinal detachment. 2 months later, the patient had no light perception with RE phthisis. LE VA was 20/33, and granulomatous panuveitis was observed with mutton-fat KPs, AC cells, vitreous cells, and optic disc swelling. The patient was diagnosed with SO and treatment with prednisone was instituted. Despite initially declining RE enucleation, the patient eventually proceeded with enucleation due to unbearable pain. Inflammation was controlled by the 4th month, and the patient was kept on maintenance therapy with prednisone. 2 years later, LE VA was 20/25, and the eye was quiet with few peripheral choroidal pigmentary lesions. Treatment was discontinued, and the patient was monitored for 4.5 years with a favorable outcome. At the last follow-up, 7 years after presenting symptoms and while treatment-free, the patient’s LE VA was preserved on 20/28.

Case 4

**Fig. 5:**
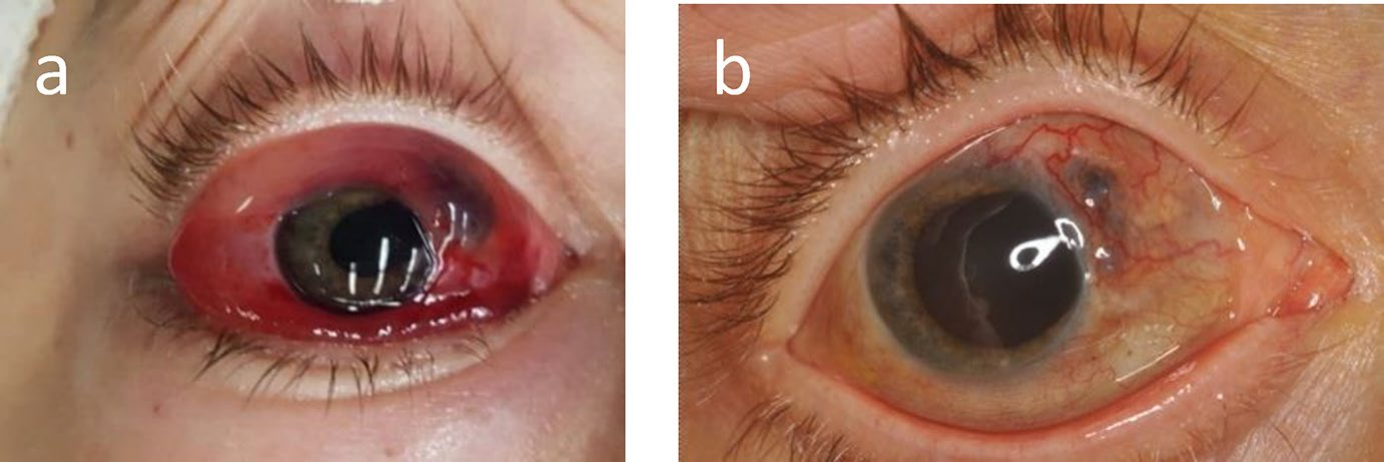
**a** A frontal photograph of the inciting right eye showing chemosis, scleral perforation with iris prolapse, and corectopia. **b** A well-sealed scleral wound is noted following primary repair

A 34-year-old female patient with Turner Syndrome, presented within 12 h post blunt trauma with a ruptured RE sclera, prolapsed iris, corectopia, chemosis, and hyphema (Fig. 5a). VA was RE 20/133 and LE 20/25. The retina was intact, and ultrasonography confirmed the findings. The patient underwent primary wound repair. 8 days later, RE vision improved to 20/66 and the scleral wound was sealed (Fig. 5b). Fundus examination was normal.

**Fig. 6:**
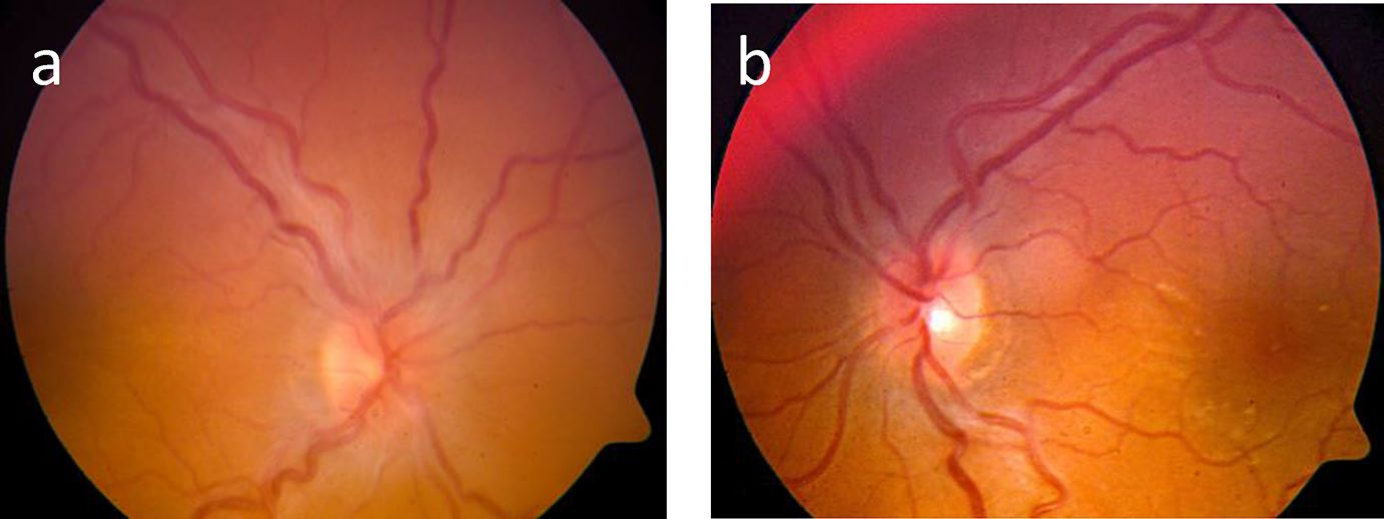
Color fundus photography of both eyes. **a** On the left, the right eye has a hazy fundus view and blurry optic disc borders. **b** On the right, the left fundus is clearly observed with normal-looking optic disc and a blunt foveal reflex

**Fig. 7:**
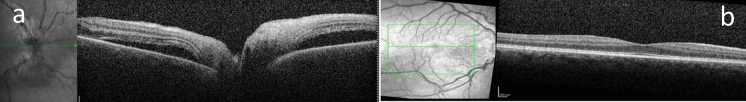
**a** OCT of the right eye shows exudative retinal detachment on both sides of the optic disc. **b** OCT shows normal foveal contour with resolved subretinal fluid

**Fig. 8:**
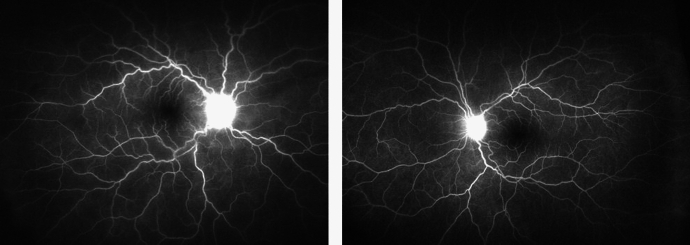
Fluorescein angiography shows bilateral optic disc hyperfluorescence

4 weeks later, the patient presented with headache, redness and decreased vision in LE. BCVA was 20/50 in both eyes (BE). On examination, RE showed mutton fat KPs, AC cells (+ 3), vitreous cells (+ 3), and blurry optic disc borders (Fig. 6a). LE examination showed mutton fat KPs, AC cells (+ 3), and a blunt foveal reflex (Fig. 6b). OCT revealed RE ERD (Fig. 7a) and FFA showed optic disc leakage in BE (Fig. 8). The patient was diagnosed with SO and she was promptly treated with a 5-day-pulse of intravenous methylprednisolone. Also, the patient underwent successful pericardial patch grafting in order to reinforce the scleral wound at the site of the previous trauma. Subsequently, 4 months later, the patient developed LE ERD. ERD resolved bilaterally after 6 weeks of treatment with prednisone and azathioprine.

One year and 8 months after the initial presentation with SO, prednisone was discontinued, and 6 months later, azathioprine was also discontinued. At the last follow-up visit, 3 years and 4 months after achieving drug-free remission and 5.5 years after the first presentation, the patient showed no signs of active inflammation and maintained a final BCVA of 20/50 in BE. OCT revealed no subretinal fluid (SRF) in either eye **(**Fig. 7b**).**

Case 5

A 57-year-old female patient with a history of DM Type 2, hypertension (HTN), and dyslipidemia sustained a perforating eye injury of the initially blind LE (secondary to trauma in childhood). 6 months later, she presented with RE blurred vision of 5-day-duration.

**Fig. 9:**
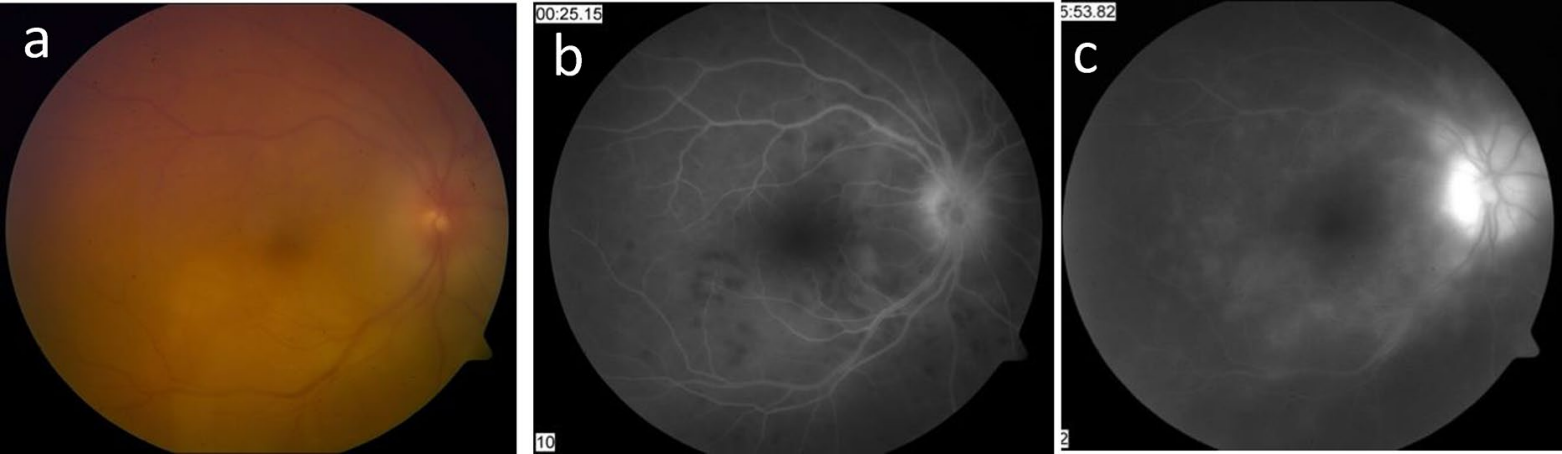
**a** Color fundus photography of the right eye shows a hazy view secondary to vitritis, optic disc edema and choroidal lesions along the inferotemporal arcade. **b** Fluorescein angiography of the right eye shows hypofluorescent lesions in the early phase and **c** marked leakage of the posterior pole lesions in the late phase with marked optic disc leakage

On presentation, LE was phthisic with NLP. RE BCVA was 20/40 with active granulomatous panuveitis, faint choroidal lesions along the inferotemporal arcade and optic disc edema (Fig. 9a). Diffuse hypofluorescent lesions were noted in the arterial-venous phase, with significant leakage in the late phase and marked optic disc leakage (Fig. 9b and c).

The patient was diagnosed with sympathetic ophthalmia. Systemic and topical steroids were initiated and LE evisceration was completed 2 days later. RE VA improved to 20/28.

5 months after presentation with SO, the patient complained of RE blurred vision. BCVA was 20/125. Examination revealed relapse of panuveitis despite being treated with prednisone and azathioprine. Infliximab (300 mg every 4 weeks after the loading dose) was initiated. However, because of partial response, it was replaced by adalimumab (40 mg every other week by subcutaneous injection). Inflammation was finally controlled 2 years after presentation with triple immunosuppression: mycophenolate mofetil, prednisone and adalimumab.

**Fig.10:**
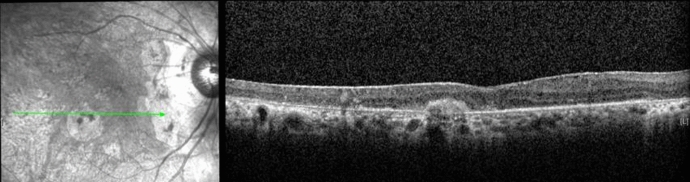
OCT of the right eye six years after presentation shows thinning of retinal layers and scars at the level of retinal pigment epithelium

**Fig. 11:**
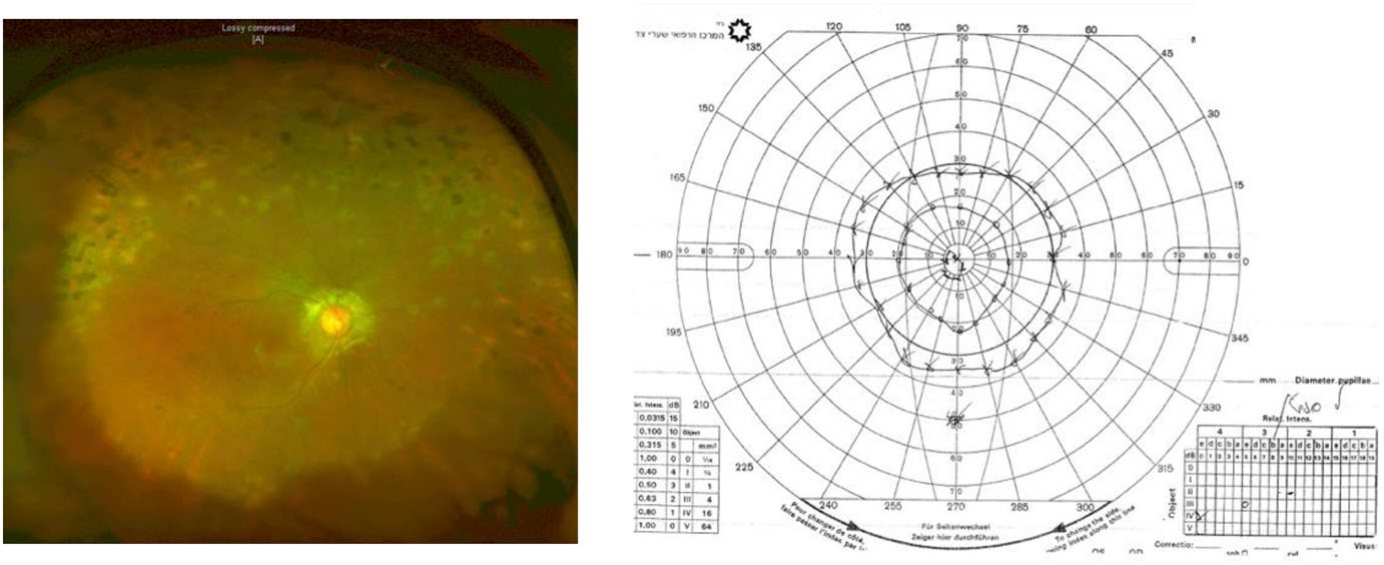
**a** Pseudo-color fundus photo of the right eye shows clear vitreous, peripapillary scarring, and peripheral hypo and hyperpigmented choroidal lesions. **b** The visual field of the RE shows a constricted field of 30 degrees at maximal light intensity (4dB) and 20 degrees at less light intensity (3dB)

In the last follow-up visit, 6 years after presentation, RE BCVA was 20/100 and OCT showed marked retinal thinning and scarring (Fig. 10). Peripheral retinochoroidal atrophic scars and peripapillary scarring were noted (Fig. 11a). Unfortunately, the patient had a constricted visual field, as seen in Fig. 11b.

Case 6

A 42-year-old female patient with DM Type 2, obesity, and hypertension presented with RE decreased vision and redness. She had a history of LE perforating trauma in the LE 30 years earlier, and LE vision had been NLP since then. On examination, LE appeared phthisic, and RE had signs of dust-like keratic precipitates, AC cells (+ 1) and flare (+ 1), optic disc edema, and diffuse punched-out choroidal lesions compatible with Dalen-Fuchs nodules, which were later confirmed with OCT and FFA imaging. RE BCVA was 20/25. Other causes of ocular inflammation were ruled out through laboratory workup, including sarcoidosis, syphilis, and tuberculosis. The patient was diagnosed with SO and treated with topical steroids, prednisone and MTX, which partially controlled the inflammation. However, after experiencing prolonged pain and discomfort in the LE, the patient underwent LE enucleation. Attempts to decrease systemic steroids led to an exacerbation of inflammation. As a result, chronic anterior uveitis led to posterior synechiae and posterior subcapsular cataract formation. Additional ocular complications included peripheral choroidal pigmentary lesions and maculopathy. In the last follow-up visit, 3 years after the presentation, VA in RE was 20/40. The patient was then lost to follow-up.

Case 7

A 76-year-old male patient with a history of DM type 2 underwent RE cataract surgery in another hospital which was complicated by Pseudomonas endophthalmitis. This resulted in several vitrectomies, which led to phthisis bulbi, and NLP. A year later, the patient complained of progressively decreased vision in his LE. Upon initial assessment, the patient had NLP in his RE and hand motion (HM) in his LE. The LE showed hyperemic conjunctiva and mutton-Fat KPs, flare, and cells (+ 3) in the AC, hazy fundus view with vitreous cells (+ 3), and multiple DF nodules. LE OCT showed macular edema and FFA showed diffuse late leakage and hyperfluorescent optic disc.

The patient was diagnosed with SO. He was treated with a pulse of intravenous methylprednisolone for 3 days, and then prednisone and mycophenolate mofetil were initiated. Macular edema resolved, and panuveitis was resolving. Subsequently, the patient developed acute pancreatitis due to gallbladder stones, leading to the discontinuation of systemic IMT. Due to the patient’s health status and affected liver function tests, systemic steroids and mycophenolate mofetil treatment was not resumed, and the patient was lost to follow-up.

2 years later, the patient presented with an exacerbation of SO. On examination, signs of iritis were noted with posterior synechiae, rubeosis, and mature cataract. The patient was treated with intravenous methylprednisolone, but inflammation was only partially controlled. At his last follow-up, 3 years after the onset of symptoms, LE VA was LP. The patient was then lost to follow-up.

## Discussion

This report provides information on seven patients with SO, including their characteristics and long-term clinical outcomes. It aligns with previous studies, indicating that ocular trauma is the primary risk factor for SO, accounting for 75–80% of all SO cases [13]. In our review, 6 out of 7 patients (86%) experienced ocular trauma as the main trigger. While intraocular surgeries are not a common cause of SO, they have become a prevalent risk factor. Kilmartin et al. [14] reported that the risk of SO after pars plana vitrectomy and external retinal detachment repair was 1 in 1152 retinal surgical procedures. Additionally, as observed in our study, the majority of patients with SO were males, which is due to the fact that ocular injuries are more prevalent among males. According to Park et al., [15] ocular injuries caused by major trauma are more common in men, with an incidence rate of 81.7%. Moreover, most SO cases mentioned in the literature report symptoms occurring within one year after the incident, as observed in our study [10, 16]

Even though there is no consensus on SO treatment, it is widely recognized by experts that recognizing and treating the condition promptly is crucial. The key to controlling inflammation and maintaining good visual outcomes is initiating immunosuppressive treatment using systemic steroids [17]. However, steroid therapy has risks, including cataract formation, diabetes mellitus, hypertension, adrenal insufficiency, and osteoporosis [18]. Jonas et al. [19] reported on a patient with SO who developed severe Cushing’s disease after long-term treatment with systemic steroids, including steroid-induced diabetes mellitus, arterial hypertension, and obesity. To improve the prognosis, a combination of steroids, antimetabolites [20], or biologic immune modulators [21] is now commonly used at the onset of the disease. In our study, all patients received systemic steroids at presentation, and in six cases (86%), MTX, Azathioprine, mycophenolate mofetil, and TNF-α inhibitors were added to control inflammation.

Several studies have discussed and debated the option of preventive enucleation of the exciting eye. The prevailing theory suggests that enucleation within 2 weeks of the initial trauma can prevent SO [22]. However, controversy still exists on whether enucleation is still beneficial once SO symptoms have begun. According to Lubin et al., [5] enucleation within 2 weeks of symptom onset can improve visual outcomes in the sympathizing eye.

In our small study, no correlation was seen between the visual outcome and the time of enucleation or evisceration. Patient #2 had enucleation within 2 weeks of the onset of symptoms and preserved initial BCVA, whereas patient #5 underwent evisceration within 2 days of symptom onset and experienced a decrease in initial BCVA. However, our findings suggest that it was unclear whether enucleation or evisceration improves the visual outcome of SO, as we have limited data. Nowadays, ophthalmologists are less likely to recommend enucleation as a precaution, as SO is rare, and the affected eye may eventually recover. We observed this in Case 4, who maintained the same BCVA in her exciting eye 7 years after the initial assessment. Therefore, enucleation should only be considered in cases with no potential for vision improvement.

Patients with SO can experience future exacerbations in the sympathizing eye [18]. Previous studies have shown that treatment cessation may have implications for this. Patel et al. [4] found that 25% of their patients relapsed after stopping treatment, with a mean post-cessation period of 131 months. No association was found between exacerbation and age, gender, white blood cell count nadir, or duration of treatment. Gupta et al. and Makley et al. reported that a significant number of patients experienced recurrences or flares of their condition after every attempt to stop steroid treatment [16, 23]. In our study, five patients had a relapsing nature. Three of them (pt#2, 4, and 6) experienced a flare-up after stopping or reducing steroid treatment, and two (pt#5 and 7) experienced flare-ups while on maintenance treatment with systemic steroids. Hence, it is difficult to determine the connection between relapse and stopping treatment. Nevertheless, long-term immunosuppressive therapy is recommended for visual preservation [24].

Nearly half of the patients with SO reportedly have a BCVA of 20/40 or less in their sympathizing eye [17]. In our study, the final BCVA in the sympathizing eye was 20/40 or more in 4 cases (57%). We found a link between the patient’s health status, age, co-morbidities, and secondary complications to their visual outcome. Patients 1–3 were young, healthy, and had a favorable long-term visual condition (Tables 1, 2). Case 4 was 34 years old with Turner syndrome but no health issues at the time of the disease and maintained initial BCVA in BE. On the other hand, cases 5–7 had co-morbidities (DM/HTN/Hyperlipidemia) that may have negatively influenced their visual outcome.

One theory is that controlling blood glucose levels is challenging for diabetic patients on steroid treatment, leading to noncompliance and uncontrolled uveitis or its reactivation. Another hypothesis is that chronic hyperglycemia increases the production of pro-inflammatory cytokines [25], potentially intensifying and worsening inflammatory conditions such as in SO. For instance, studies have suggested that diabetes can worsen outcomes in patients with systemic lupus erythematosus (SLE), causing an increased risk of cardiovascular disease, renal damage, and other complications [26]. More recent data suggested that individuals with diabetes and multiple sclerosis (MS) may experience a more aggressive disease course due to intensified oxidative stress [27]. However, this hypothesis has not been explored in previous publications, and due to the limited number of cases, we cannot confirm or deny its validity. As a result, further research is necessary to investigate this topic.

Complications in patients with SO may include glaucoma, cataract formation, choroidal atrophy, maculopathy, ERD, macular edema, and phthisis bulbi [21]. The frequency of these complications varies among different studies. According to Galor et al., [28] 47% of patients experienced complications at presentation, with 40% developing new complications each year. Makley et al. [16] reported that 70% of cases had sequelae, with cataract formation (47%), secondary glaucoma (43%), ERD (25%), and severe choroidal atrophy (25%) being the most common. In our review, 4 cases (57%) showed complications at presentation, with ERD in the sympathizing eye in 2 cases (29%) and phthisis bulbi in the exciting eye in 4 cases (57%). Cases 1, 2, and 3, only had choroidal atrophic lesions, while Case 4 had additional complications of pigmentary changes in the macula. Cases 5–7 had more severe complications such as posterior synechiae, maculopathy, cataracts, and rubeosis. These results reinforce the hypothesis that pre-existing conditions such as diabetes can worsen inflammation control, resulting in more complications and a less positive visual outcome. This suggests that a patient’s age and overall health can have a direct impact. However, further studies are needed as our review was limited to a small number of patients.

Our study is unique as we have followed up the patients for a mean of 6.8 years between 2002 and 2022, which is longer than most other studies. While Galor et al. and Gupta et al. have reported on long-term outcomes, their follow-up durations were shorter than ours [23, 28]. Makley et al. [16] reported a mean follow-up of 10.6 years. Our extended follow-up is particularly important as SO is an ongoing process with recurrent exacerbations and complications. Therefore, it was crucial to record the clinical outcomes over several years in our review.

Insights into the follow-up periods of patients with SO have been provided in previous articles [16, 23, 28]. However, there seems to be a lack of direct mention concerning the duration after discontinuing treatment and the timeframe during which patients remained without a relapse. Our report calculates a mean (± SD) follow-up period of 28 ± 22.8 months in four patients (pt#1–4) after a drug-free remission period. This information is particularly valuable as it raises an important question about whether patients can successfully discontinue therapy. By further researching the duration of follow-up periods after discontinuing treatment, healthcare professionals can make more informed decisions about the management of SO.

## Conclusion

Sympathetic Ophthalmia is a severe sight-threatening condition that follows an ocular trauma or intraocular surgery. The disease responds well to prompt diagnosis and treatment, and recurrences are expected. Since this disease is rare, it is difficult to study, but more research is needed to understand how different treatments and their duration affect the condition. In this study, younger, healthier individuals had better visual outcomes, while older patients with co-morbidities had poorer outcomes. To prevent a relapse, long-term therapy is important, and some cases may require additional use of biological or immunomodulatory agents.
